# T Lymphocyte-Derived Exosomes Transport MEK1/2 and ERK1/2 and Induce NOX4-Dependent Oxidative Stress in Cardiac Microvascular Endothelial Cells

**DOI:** 10.1155/2022/2457687

**Published:** 2022-09-28

**Authors:** Filip Rolski, Marcin Czepiel, Karolina Tkacz, Katarzyna Fryt, Maciej Siedlar, Gabriela Kania, Przemysław Błyszczuk

**Affiliations:** ^1^Department of Clinical Immunology, Jagiellonian University Medical College, Cracow, Poland; ^2^Center of Experimental Rheumatology, Department of Rheumatology, University Hospital Zurich, Switzerland

## Abstract

**Background:**

Activation of endothelial cells by inflammatory mediators secreted by CD4^+^ T lymphocytes plays a key role in the inflammatory response. Exosomes represent a specific class of signaling cues transporting a mixture of proteins, nucleic acids, and other biomolecules. So far, the impact of exosomes shed by T lymphocytes on cardiac endothelial cells remained unknown.

**Methods and Results:**

Supernatants of CD4^+^ T cells activated with anti-CD3/CD28 beads were used to isolate exosomes by differential centrifugation. Activation of CD4^+^ T cells enhanced exosome production, and these exosomes (CD4-exosomes) induced oxidative stress in cardiac microvascular endothelial cells (cMVECs) without affecting their adhesive properties. Furthermore, CD4-exosome treatment aggravated the generation of mitochondrial reactive oxygen species (ROS), reduced nitric oxide (NO) levels, and enhanced the proliferation of cMVECs. These effects were reversed by adding the antioxidant apocynin. On the molecular level, CD4-exosomes increased NOX2, NOX4, ERK1/2, and MEK1/2 in cMVECs, and ERK1/2 and MEK1/2 proteins were found in CD4-exosomes. Inhibition of either MEK/ERK with U0126 or ERK with FR180204 successfully protected cMVECs from increased ROS levels and reduced NO bioavailability. Treatment with NOX1/4 inhibitor GKT136901 effectively blocked excessive ROS and superoxide production, reversed impaired NO levels, and reversed enhanced cMVEC proliferation triggered by CD4-exosomes. The siRNA-mediated silencing of *Nox4* in cMVECs confirmed the key role of NOX4 in CD4-exosome-induced oxidative stress. To address the properties of exosomes under inflammatory conditions, we used the mouse model of CD4^+^ T cell-dependent experimental autoimmune myocarditis. In contrast to exosomes obtained from control hearts, exosomes obtained from inflamed hearts upregulated NOX2, NOX4, ERK1/2, MEK1/2, increased ROS and superoxide levels, and reduced NO bioavailability in treated cMVECs, and these changes were reversed by apocynin.

**Conclusion:**

Our results point to exosomes as a novel class of bioactive factors secreted by CD4^+^ T cells in immune response and represent potential important triggers of NOX4-dependent endothelial dysfunction. Neutralization of the prooxidative aspect of CD4-exosomes could open perspectives for the development of new therapeutic strategies in inflammatory cardiovascular diseases.

## 1. Introduction

Cardiovascular diseases are the leading cause of morbidity and mortality in the world, and coronary heart disease is the most common form of cardiovascular disease [1]. Chronic oxidative stress in endothelial cells that leads to their dysfunction plays a key role in the pathogenesis of atherosclerosis and coronary microcirculation dysfunction [2, 3]. More recently, endothelial dysfunction was implicated in life-threatening complications in COVID-19 patients [4].

The endothelial barrier is composed of vascular endothelial cells, endothelial glycocalyx, and basement membrane. The main function of this barrier is not only the separation of blood from underlying tissues but also the control of nutrient delivery, metabolic homeostasis, prevention of thrombotic events, management of immune cell trafficking, and regulation of blood pressure. In a healthy condition, the production of nitric oxide (NO) exerting vasodilating properties represent a particularly important aspect of endothelial cell activity. Loss or impairment of physiological properties of the endothelium is termed endothelial dysfunction [5]. Oxidative stress is characterized by an increase of reactive oxygen species (ROS), which include hydrogen peroxide (H_2_O_2_), superoxide anion (O_2_^–^), and hydroxyl radicals. Increased ROS production together with reduced endothelial nitric oxide synthase (eNOS) activity and NO bioavailability plays a pivotal role in endothelial dysfunction [6]. ROS serve as signal transmitters regulating numerous cellular processes, and their excessive production affects cellular metabolism, transcriptomic profile, and signaling pathway activities leading to abnormal cellular function [7, 8]. In endothelial cells, ROS are produced by several cellular sources including mitochondria, membrane-bound NADPH oxidases (NOX) 1-5, and other enzymes, such as oxidases, peroxidases, cytochromes, mono- and dioxygenases, and uncoupled eNOS. Regulation of the NOX expression is not completely understood. It involves a complex interplay between several transcription factors, co-activators/repressors, nuclear receptors, and epigenetic mechanisms [9].

Inflammation has been implicated in the activation of endothelial cells. Chronic inflammatory conditions can affect coronary microvascular function and contribute to the development of myocardial ischemia and cardiovascular events. Furthermore, chronic, low-grade inflammation of the arterial wall is a typical feature of atherosclerosis [3]. In response to inflammation, endothelial cells upregulate adhesion molecules to interact with circulating leucocytes and downregulate endothelial junctional structures to increase vascular permeability. A growing body of evidence indicates that inflammation affects ROS/NO balance in endothelial cells leading to endothelial dysfunction [8]. The inflammatory response in chronic inflammatory diseases is orchestrated mainly by cells of myeloid lineage and T lymphocytes. Activation of naïve T lymphocytes through T cell receptor (TCR) leads to expansion of the effector pool of antigen-specific T lymphocytes. Activated naïve T cells turn into effector T cells, and antigen-presenting cells direct their polarization into the specific T helper subtype [10]. TCR activation results in the production and secretion of several primary inflammatory cytokines and chemokines, which often exaggerate inflammatory response.

Mid- and long-distance cell-to-cell interaction is not limited to secretory proteins but can also be mediated by extracellular vesicles, which are shed by one class of cells and absorbed by others [11]. Extracellular vesicles represent fragments of the cytoplasm that carry molecular cargo that includes membrane and cytosolic proteins, lipids, and various RNAs. Practically, all cells actively release various types of membrane vesicles of endosomal and plasma membrane origin in the process of exocytosis. Extracellular vesicles are classified by size and are usually separated by differential centrifugation. The larger size class called microvesicles is heterogeneous (200 to ~1,500 nm), while the smaller size class called exosomes is relatively homogeneous in size (50 - 150 nm). Exosomes bear specific surface markers and adhesion molecules, transport various lipids, proteins, mRNAs, and microRNAs, and therefore have to be considered as a separate class of immunomodulatory molecules [11]. Extracellular vesicles have been demonstrated to play a modulatory role in various cardiovascular disorders. So far, the proinflammatory potential of T cell-derived exosomes has not been extensively investigated. In this work, we specifically analyzed the effect of exosomes produced by activated T lymphocytes in the context of endothelial cell activation.

## 2. Materials and Methods

### 2.1. Experimental Autoimmune Myocarditis (EAM) Model

EAM was induced in 6–8 week-old Balb/c mice by subcutaneous injection of 200 *μ*g of *α*-MyHC_614-634_ peptide (Ac-RSLKLMATLFSTYASADR-OH, Caslo, Denmark) emulsified in 1 : 1 ratio with complete Freund's adjuvant (Difco, USA) at days 0 and 7. Mice were anesthetized by intraperitoneal injection of ketamine (75 mg/kg) and euthanized by cervical dislocation on day 21. All experiments were performed in accordance with Polish law and were approved by local authorities (license number 234/2019). Animal experiments followed the guidelines of Directive 2010/63/EU of the European Parliament on the protection of animals used for scientific purposes.

### 2.2. Isolation and Culture of Cardiac Microvascular Endothelial Cells

Preliminary experiments were performed using commercially available primary cardiac microvascular endothelial cells (cMVECs) from Balb/c mice (Cedarlane, Canada). Further experiments were performed on primary cMVECs isolated from 4 weeks old Balb/c mice. Briefly, mice were euthanized, and hearts were perfused with ice-cold PBS supplemented with 5 mM EDTA. Three hearts were cut into small pieces, suspended in 1 ml of RPMI 1640 (Corning, USA) supplemented with 50 *μ*g/ml Liberase TL (Roche, Switzerland), and incubated at 37°C. Every 10 minutes, hearts were pipetted until tissues were completely disintegrated. Cell suspension was filtered through 70 *μ*m and 40 *μ*m cell strainers and centrifuged for 1 minute at 400 g. Single cell suspension was resuspended in the growth medium: RPMI 1640 containing 10% fetal bovine serum (FBS; USA, Gibco), 0.5 ng/ml mouse recombinant endothelial growth factor (BioLegend, USA), and 1 ng/ml mouse recombinant fibroblast growth factor (BioLegend, USA). Cells isolated from a single mouse heart were seeded in a 10 cm cell culture dish (Falcon, USA) coated with 0.2% gelatine and incubated at 37°C for 60 minutes. Next, nonadhesive cells were removed, and cMVECs were cultured in the growth medium and were passaged after reaching at least 90% confluence. The purity of all cultures was confirmed by CD31 staining using flow cytometry (over 90% of CD31^+^ cells). Cells between passages 3 and 7 were used in the experiments. In selected experiments, the cMVECs were treated with 50 *μ*M apocynin (Sigma, USA), 2 *μ*M GKT136901 (NOX inhibitor, Merck, Germany), 5 *μ*M FR180204 (ERK inhibitor, Tocris Bioscience, GB), or 5 *μ*M U0126 (MEK inhibitor, Tocris Bioscience, GB). Apocynin, inhibitors, and exosomes were added at the same time. All treatments were performed 1 day after cMVECs reached full confluence.

### 2.3. Isolation and Culture of CD4^+^ T Lymphocytes

CD4^+^ T lymphocytes were isolated from spleens of 4-6 weeks old Balb/c mice. Briefly, spleens were mechanically mashed through a 70 *μ*m cell strainer following erythrocyte lysis using the ammonium-chloride-potassium buffer, and the obtained cell suspension was filtered through a 40 *μ*m cell strainer. CD4^+^ T cells were isolated using Dynabeads Untouched Mouse CD4 Cells Kit (ThermoFisher, USA) according to the manufacturer's instructions. Isolated CD4^+^ T cells were cultured in RPMI 1640 supplemented with 10% FBS. The medium used in T cell cultures was depleted from exosomes by 1-hour centrifugation at 100 000 g. T cell activation was performed with Mouse T-Activator CD3/CD28 Dynabeads (ThermoFisher, USA). After 3 days of culture (density about 4 × 10^6^ T cells/ml), the medium was collected for exosome isolation or used as conditional medium.

### 2.4. Isolation and Measurement of Exosomes

CD4^+^ T cell-derived exosomes were isolated from conditioned medium of activated CD4^+^ T cells as described previously [12]. Heart exosomes were isolated from mice hearts with induced EAM and healthy age-matched mice. Each heart was perfused with ice-cold PBS, cut into small pieces, and suspended in 1 ml of RPMI 1640 (Corning, USA) supplemented with 50 *μ*g/ml Liberase TL and incubated at 37°C for approximately 40 minutes. Tissue pieces were pipetted every 10 minutes until single-cell suspension was obtained. Liberase TL was neutralized by the addition of 2 ml RPMI 1640 containing 10% FBS. The suspension was further diluted to 10 ml with PBS and centrifuged for 5 minutes at 4 000 g. To obtain exosomes, conditioned medium or collected supernatants were centrifuged at 20 000 g for 30 minutes to remove cell debris. Next, obtained supernatants were centrifuged for 1 hour at 100 000 g, and pellets were resuspended in 100 *μ*l PBS and stored at -20°C for up to a month. Exosomes were used at concentration of 10^8^ particles/ml unless otherwise stated. 10^8^ particles/ml represented a typical concentration of exosomes in medium conditioned of activated T lymphocytes. Exosomes were measured using the NanoSight tracking analysis (NTA) system: the LM10HS microscope was equipped with the LM14 488 nm laser module (Malvern Instruments Ltd., Malvern, UK). Samples were diluted in PBS to provide counts within the detection range of the instrument. One-minute duration videos were recorded for each batch of exosomes. Particle movement was analyzed with the NTA 3.1 NanoSight software according to the manufacturer's protocol.

### 2.5. Exosome Labeling

Isolated exosomes were labeled with PKH26 Red Fluorescent Cell Linker Kit (Sigma-Aldrich) according to manufacturer's protocol and washed and resuspended in PBS. cMVECs were grown to full confluence on 24-well plates and treated with 4x10^8^ PKH26 labeled exosomes for 18 h, washed with PBS, and resuspended in growth medium. The immunofluorescence of live cells was analyzed using an Olympus BX53 microscope equipped with Olympus XC50 camera (Olympus, Tokyo, Japan).

### 2.6. Flow Cytometry

Cells were cultured in 24-well plates coated with 0.2% gelatin. Before the experiment medium was removed, cells were rinsed 3 times with PBS, collected using trypsin, and washed 2 times in PBS. Total ROS production was measured by staining with 0.5 ml of 10 *μ*M 2′,7′-dichlorofluorescein diacetate (H_2_DCFDA, ThermoFisher, USA) diluted in PBS in the dark at 37°C for 10 minutes. The superoxide production by mitochondria was evaluated using the MitoSOX™ Red reagent (ThermoFisher, USA). cMVECs were suspended in 0.25 ml growth medium containing 2 *μ*M MitoSox and incubated in the dark at 37°C for 30 minutes. Nitric oxide levels were determined with 4,5-diaminofluorescein diacetate (DAF2 DA, Abcam, UK). Cells were incubated in PBS containing 5 *μ*M DAF2 DA in the dark at 37°C for 20 minutes. To perform membrane adhesion molecules measurements, cultured cMVECs were labeled with anti-ICAM1-PE (1 : 500, clone YN1/1.7.4, Thermo Fisher, USA), anti-VCAM1-PE-Cy7 (1 : 500, clone 429, Biolegend, USA) anti-P-selectin-APC (1 : 500, clone RMP-1, BioLegend, USA), and anti-CD31-FITC (1 : 500, clone 390, ThermoFisher, USA) using standard procedure. Dead cells were determined using propidium iodide (ThermoFisher, USA) staining and excluded from the analysis. After each respective staining, cells were washed with growth medium, centrifuged at 300 g for 5 minutes, washed again with PBS, and resuspended in flow cytometry buffer (2% FBS, 1 mM EDTA in PBS). All samples were acquired using the BD FACSCanto II analyzer (BD Biosciences), and the data were analyzed with the FlowJo software (Tree Star, FlowJo X 10.0.7., USA).

### 2.7. Peroxynitrite Measurement

Production of peroxynitrite was determined using Cell Meter™ Fluorimetric Intracellular Peroxynitrite Assay Kit ∗Green Fluorescence∗ (ATT Bioquest, USA). Briefly, cMVECs were seeded onto 96-well plate coated with 0.2% gelatine. The dye was diluted according to manufacturer instructions in growth medium. Cells were incubated for 30 minutes in the dark at 37°C in 50 *μ*l of staining solution. Cells were washed twice with PBS, and fluorescence was measured using M200 PRO plate reader (TECAN Instruments, Switzerland).

### 2.8. Proliferation Assay

5000 cMVECs per well in 0.2 ml of growth medium were seeded onto 96-well plates coated with 0.2% gelatin. Cells were stimulated with T cell-derived exosomes alone or in combination with 50 *μ*M apocynin, 2 *μ*M GKT136901, NOX inhibitor, 5 *μ*M FR180204, or 5 *μ*M U0126. Cell proliferation was measured after 2 days using CyQUANT™ Cell Proliferation Assay (ThermoFisher, USA) according to the manufacturer's instructions.

### 2.9. Adhesion Assay under Shear Stress

The BioFlux 200 48-well plate 0-20 dyn (Fluxion, USA) microcapillaries were coated with 1% gelatin (ThermoFisher, USA) and 25 *μ*g/ml human fibronectin (ThermoFisher, USA) solution in growth medium for 24 h at 37°C. Single cell suspension of MVECs (2.5 × 10^6^/ml) in growth medium was loaded into capillaries using the BioFlux 200 system (Fluxion, USA), and cells were cultured inside the microcapillaries until the formation of a confluent monolayer. Cells were stimulated with either 5 ng/ml recombinant mouse TNF-*α* (BioLegend), T cell-derived exosomes, or a combination of both for 16 hours. For the adhesion assay, single cell suspension of freshly isolated mouse splenocytes was labelled with the CellTrace CFSE (ThermoFisher, USA) and used at concentration of 3 × 10^5^ cells/ml. The flow of splenocytes at 1 dyn/cm^3^ through the microcapillaries coated with cMVECs was induced for 30 minutes, and adhesion of CFSE-labeled splenocytes was analyzed with a fluorescence microscope (LS720, Etaluma, USA).

### 2.10. RT-PCR

Total RNA was extracted with TRIzol (Invitrogen) according to manufacturer recommendations. 100 ng total RNA was used for reverse transcription using the NG dART RT Kit (EURx, Poland). Quantitative real-time PCR was performed using the SYBR Green PCR Master Mix (EURx, Poland) and oligonucleotides complementary to transcripts of the analyzed genes using the Quant Studio 6 Real-Time PCR system (Applied Biosystems, USA). The following oligonucleotides were used in this study: 5′-GAGCGACTCAAACTGCCCT-3′, *Mapk3* 5′-GGTTGTTCCCAAATGCTGACT-3′ and 5′-CAACTTCAATCCTCTTGTGAGGG-3′, *Map2k1* 5′-AAGGTGGGGGAACTGAAGGAT-3′ and 5′-CGGATTGCGGGTTTGATCTC-3′, *Ywhaz* 5′-GAAAAGTTCTTGATCCCCAATGC-3′ and 5′-TGTGACTGGTCCACAATTCCTT-3′, *Nox3* 5′-CAACGCACAGGCTCAAATGG-3′ and 5′-CACTCTCGTTCAGAATCCAGC-3′, *Cyba* 5′-TGCCAGTGTGATCTATCTGCT-3′ and 5′-TCGGCTTCTTTCGGACCTCT-3′, *Nox1* 5′-AGCTTTCTGAGTAGGTGTGCAT-3′ and 5′-CCCAACCAGGAAACCAGAAACA-3′, *Nox2* 5′-CCTCTACCAAAACCATTCGGAG-3′ and 5′-CTGTCCACGTACAATTCGTTCA-3′, *Nox4* 5′-GAAGGGGTTAAACACCTCTGC-3′ and 5′-ATGCTCTGCTTAAACACAATCCT-3′, *Sod1* 5′-AACCAGTTGTGTTGTCAGGAC-3′ and 5′-CCACCATGTTTCTTAGAGTGAGG-3′, and *Sod2* 5′-CAGACCTGCCTTACGACTATGG-3′ and 5′-CTCGGTGGCGTTGAGATTGTT-3′. Transcript levels of *Ywhaz* were used as endogenous reference, and the relative gene expression was calculated using the 2^−*ΔΔC*t^ method.

### 2.11. Immunoblotting

cMVECs, CD4^+^ T lymphocytes, and exosomes were lysed in RIPA buffer supplemented with protease and phosphatase inhibitors (ThermoFisher, USA). Immunoblotting procedure was performed as described previously [13] using the following antibodies: anti-MEK1/2 (1 : 1000, clone D1A5, Cell Signaling, USA), anti-phospho-MEK1/2 (1 : 1000, clone S217/221, Cell Signaling USA), anti-Erk1/2 (1 : 1000, clone 137F5, Cell Signaling, USA), anti-phospho-Erk1/2 (1 : 1000, clone D13.14.4E, Cell Signaling USA), anti-eNOS antibody (1 : 1000, polyclonal, Invitrogen, USA), anti-phospho-eNOS Thr495 (1 : 1000, polyclonal, Invitrogen, USA), anti-phospho-eNOS Ser1177 (1 : 1000, polyclonal, Invitrogen, USA), anti-NOX2 (1 : 1000, clone ARC0181, Invitrogen USA), anti-NOX4 (1 : 500, clone SY0214, Invitrogen, USA), *β*-tubulin (1 : 1000, clone 228.33, Invitrogen, USA), goat anti-mouse IgG (H + L)-HRP (ThermoFisher, USA), and goat anti-rabbit IgG (H + L)-HRP (ThermoFisher, USA). The protein signal was detected using the Western Blotting Substrate (ThermoFisher, USA) and imaged with the ChemiDoc instrument (Bio-Rad, ChemiDoc Imaging System, USA). Results were analyzed with the ImageJ software (Version 1.52a, NIH, Bethesda, USA). Protein abundance was normalized to *β*-tubulin levels. To detect eNOS dimers, cell lysates were incubated in Laemmli buffer without 2-mercaptoethanol at 37°C for 5 min. For the monomer control, a sample was incubated with reducing agents at 80°C for 5 minutes. The samples were subjected to low temperature SDS-PAGE. Buffers and gels were equilibrated at 4°C, samples were separated in 6% gels at 80 V for 90 minutes, and the electrophoresis was performed in the fridge.

### 2.12. *Nox4* Silencing

*Nox4* silencing in cMVECs was performed with anti-*Nox4* siRNA (m) (Santa Cruz Biotechnology, USA). cMVECs were seeded onto 24-well plates coated with 0.2% gelatine. Cells were transfected with Lipofectamine 3000 (Thermofisher) after reaching 90% confluence. Briefly, cells were incubated in 0.3 ml transfection mix (6 *μ*l Lipofectamine 3000, 100 nM anti-*Nox4* siRNA) for 24 hours. Afterwards, 0.2 ml of growth medium was added, and cells were stimulated with CD4-derived exosomes for another 24 hours. Cells transfected with Lipofectamine 3000 without siRNA were used as controls. Optimalization of transfection was performed using Control siRNA (FITC Conjugate)-A (Santa Cruz Biotechnology, USA).

### 2.13. Histology

Mouse hearts were fixed in 4% formalin and embedded in paraffin. Standard hematoxylin/eosin staining was performed to visualize and grade the size of leukocyte infiltrates. Immunohistochemistry of CD3 cells was performed as described previously [14].

### 2.14. Statistic

Data was analyzed using Student's *t*-test or one-way ANOVA followed by Fisher's least significant difference (LSD) *post hoc* test. Differences were considered statistically significant for *p* < 0.05. All analyses were performed with GraphPad Prism 6 software (San Diego, USA), and values are expressed as mean with standard deviation (SD) as described in figure legends.

## 3. Results

### 3.1. Exosomes Shed by Activated CD4^+^ T Lymphocytes Induce Endothelial Dysfunction but Do Not Affect the Adhesive Properties of cMVECs

In the immune response, activation of CD4^+^ T cells with antigen leads to the production of proinflammatory mediators causing activation and dysfunction of endothelial cells. We could confirm that conditional medium of CD4^+^ T cells activated with anti-CD3/CD28 effectively increased membrane levels of cell adhesion antigens ICAM-1, VCAM-1, and P-selectin, induced oxidative stress in cMVECs as indicated by elevated total ROS levels, and increased mitochondrial superoxide production, and showed defective NO production (Suppl. Figure 1A).

**Figure 1:**
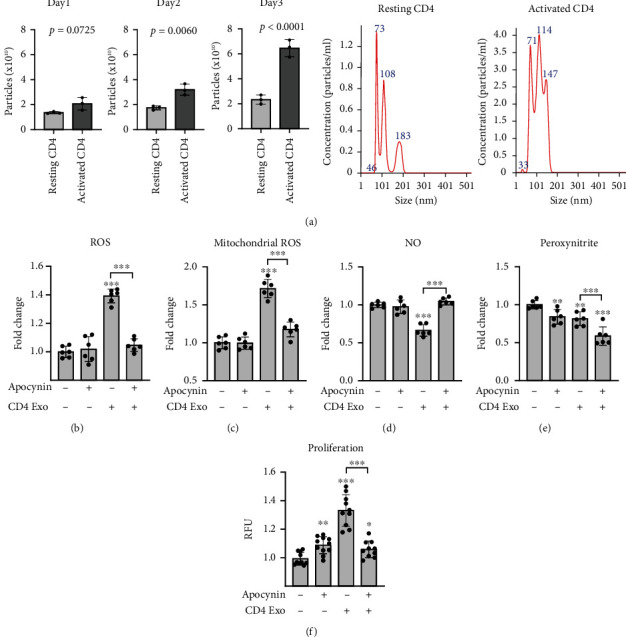
CD4-exosomes induce oxidative stress in cMVECs. Panel (a) shows quantification and representative NTA analysis of exosomes produced by resting and activated CD4^+^ T lymphocytes, *n* = 3. *p* values calculated with Student's *t*-test. Panels (b)–(e) demonstrate effect of CD4-exosomes (CD4 Exo) on cMVEC's total ROS levels (b), *n* = 6, mitochondrial ROS generation (c), *n* = 6, NO levels (d), *n* = 6, peroxynitrite levels (e), *n* = 6, and cell proliferation (f), *n* = 10, in the presence or absence of apocynin. ^∗^*p* < 0.05, ^∗∗^*p* < 0.01, and ^∗∗∗^*p* < 0.001 calculated by one-way ANOVA followed by Fisher's LSD post hoc test versus control group or for indicated groups.

**Figure 2:**
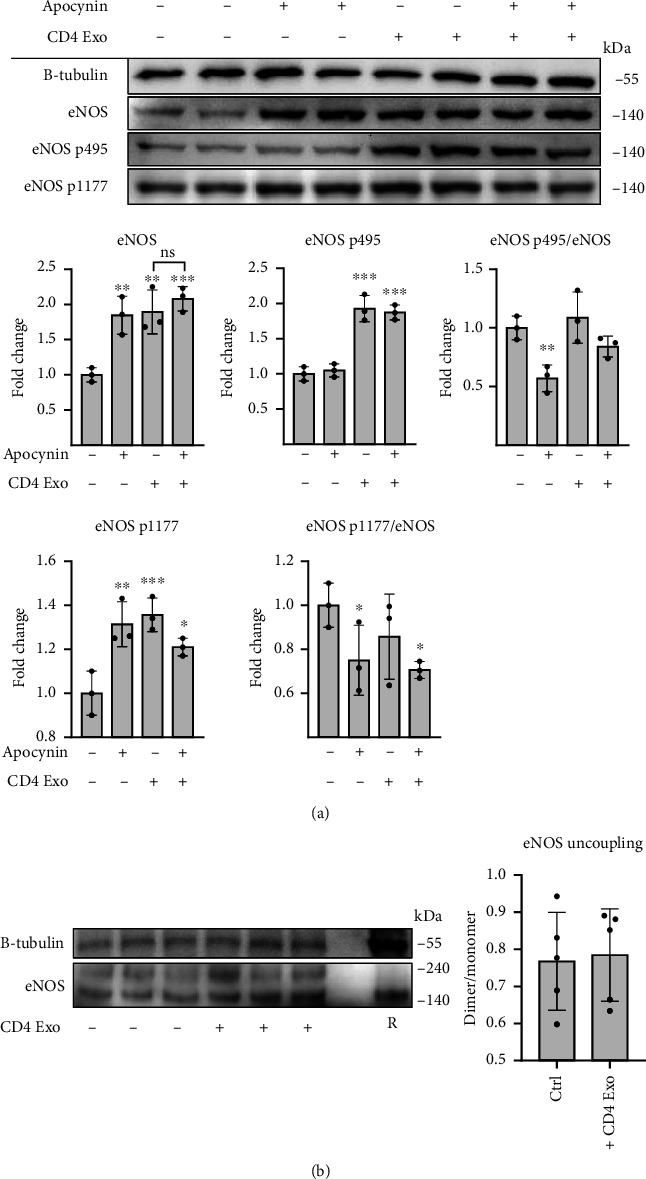
CD4-exosomes upregulate the eNOS expression in cMVECs independently of oxidative stress. Panel (a) shows representative immunoblots and densitometry of total levels of eNOS, eNOS-p495 (inhibitory site), and eNOS-p1177 (activation site) in cMVECs treated with CD4-exosomes and/or apocynin, *n* = 3. Protein levels were normalized to beta-tubulin or to total eNOS. Panel (b) shows representative immunoblots and densitometry of eNOS dimer/monomer ratio in cMVECs stimulated with CD4-exosomes, *n* = 5. *R* indicates sample with reducing agent. In each experiment, cMVECs were stimulated for 16 h. ^∗^*p* < 0.05, ^∗∗^*p* < 0.01, and ^∗∗∗^*p* < 0.001 calculated by one-way ANOVA followed by Fisher's LSD post hoc test versus control group or for indicated groups.

Activated T cells secrete not only cytokines and chemokines but also shed exosomes; therefore, we asked how these exosomes could affect the physiology of cMVECs. Activation with anti-CD3/CD28 induced secretion of exosomes by CD4^+^ T cells (Figure 1(a)). Stimulation with these CD4-exosomes (10^8^ particles/ml) for 16 hours increased ROS levels and increased mitochondrial superoxide production in cMVECs (Figures 1(b) and 1(c)). It is well known that oxidative stress can reduce NO bioavailability. Indeed, we observed reduced NO levels in cMVECs treated with exosomes (Figure 1(d)). Peroxynitrite is a highly reactive coupling product of NO and superoxide, and we observed reduced peroxynitrite levels in cells treated with CD4-exosomes (Figure 1(e)). Antioxidant apocynin can efficiently inhibit oxidative stress in endothelial cells by scavenging ROS [15, 16]. Accordingly, treatment with apocynin nearly completely protected cMVECs from CD4-exosome-induced oxidative stress and impaired NO production (Figures 1(b)–1(d)). Furthermore, treatment with CD4-exosomes enhanced cMVEC proliferation that was abolished by treatment with apocynin (Figure 1(f)). NO is produced by eNOS, which is activated by dephosphorylation at Thr495 and phosphorylation at Ser1177. Treatment with CD4-exosomes elevated total eNOS levels in cMVECs but relative phosphorylations at both sites remained unaffected (Figure 2(a)). Importantly, uncoupled eNOS represents a source of ROS rather than NO. Our data showed, however, no changes in dimer/monomer eNOS ratio in cMVECs following treatment with CD4-exosomes (Figure 2(b)). Of note, exosomes derived from resting CD4^+^ T cells lowered ROS in cMVECs and did not affect NO levels (Suppl. Figure 1B).

**Figure 3:**
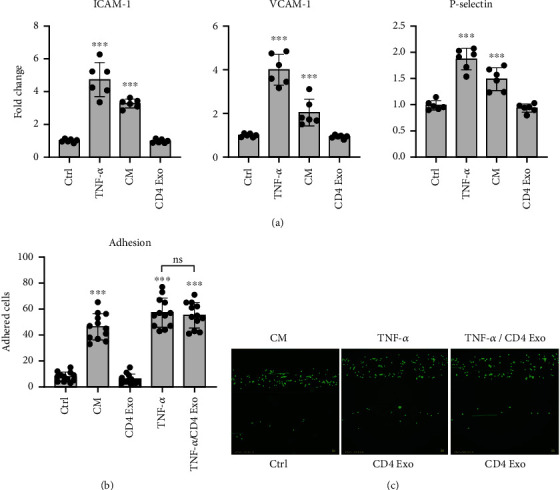
No effect of CD4-exosomes on cMVEC's adhesive properties. Panel (a) shows changes in membrane levels of adhesion molecules VCAM-1, ICAM-1, and p-selectin in cMVECs stimulated with TNF-*α*, medium conditioned of activated T lymphocytes (CM), or CD4-exosomes (CD4 Exo) for 16 h, *n* = 6. Panel (b) represents capability of treated cMVECs (for 16 h) to bind leukocytes under shear-flow conditions. Results are expressed as average number of leukocytes that firmly adhered to cMVECs after 30 minutes of shear flow, *n* = 12. Panel (c) shows representative microphotographs used in adhesion analysis. ns-*p* > 0.05, ^∗^*p* < 0.05, ^∗∗^*p* < 0.01, and ^∗∗∗^*p* < 0.001 calculated by one-way ANOVA followed by Fisher's LSD post hoc test versus control group or for indicated groups.

Oxidative stress has been associated with endothelial activation [17]. cMVECs treated with CD4-exosomes were analyzed for membrane levels of integrins involved in endothelial-leukocyte adhesion. In contrast to treatment with TNF-*α*, stimulation with CD4-exosomes did not upregulate ICAM-1, VCAM-1, and P-selectin in cMVECs (Figure 3(a)). In line with these results, the functional adhesive properties of cMVECs to bind leukocytes under shear flow were unchanged after treatment with CD4-exosomes. As expected, in activation of cMVECs with TNF-*α* induced adhesion of leukocytes, however, the addition of CD4-exosomes did not alter this response (Figure 3(b)).

### 3.2. CD4^+^ T Cell-Derived Exosomes Transport MEK1/2 and ERK1/2 and Activate NADPH Oxidases in cMVECs

**Figure 4:**
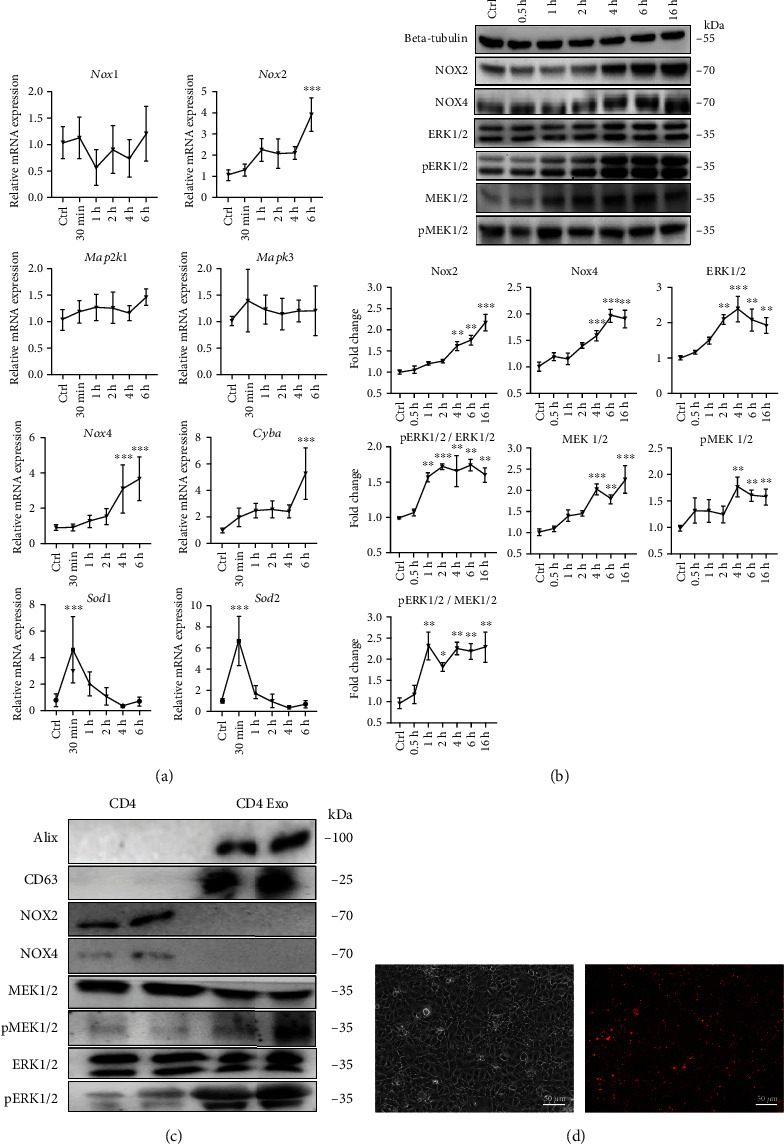
CD4-exosomes activate ERK1/2 and MEK1/2 pathway in cMVECs. Panel (a) shows changes at mRNA levels in cMVECs stimulated with CD4-exosomes for up to 6 hours, *n* = 10, for NOX4 and P22, *n* = 7, for other genes. Changes in protein levels of NOX2, NOX4, ERK1/2, ERK1/2 phosphorylation (pERK1/2), MEK1/2 and MEK1/2 phosphorylation (pMEK1/2), phosphorylation sites in cMVECs stimulated with CD4-exosomes for 16 h, and representative immunoblots are shown in panel (b), *n* = 4. Protein levels were normalized to beta-tubulin, and phosphorylated proteins were additionally normalized to their respective nonphosphorylated forms. Immunoblots of lysates (equal amounts of proteins were loaded on gel) from activated T lymphocytes (CD4) and CD4-exosomes (CD4 Exo) for indicated proteins are shown in panel (c). Panel (d) shows representative microphotographs demonstrating binding of PKH26-stained CD4-exosomes (red fluorescence) to MVECs 16 h after treatment. Scale bar = 50 *μ*m. ^∗^*p* < 0.05, ^∗∗^*p* < 0.01, and ^∗∗∗^*p* < 0.001 calculated by one-way ANOVA followed by Fisher's LSD post hoc test versus control group.

We assumed that CD4-exosome-induced oxidative stress was associated with the upregulation of endogenous NOXs in these cells. The obtained results confirmed the upregulation of *Nox2*, *Nox4*, *Cyba* (the gene that encodes P22 – a common subunit for NOX2 and NOX4 complexes), *Sod1*, and *Sod2*, but not *Nox1* at the mRNA level (Figure 4(a)). The increased levels of *Nox2* and *Nox4* in cMVECs were further confirmed at the protein level (Figure 4(b)). In the next step, we addressed the expression of transcription factors that control NOXs, such as MEK1/2 and ERK1/2. We found significant upregulation of total MEK1/2, and ERK1/2 but also p-MEK1/2 and p-ERK1/2 protein levels as early as 2-4 h after treatment with CD4-exosomes (Figure 4(b)). In contrast to the protein level, mRNA levels of *Mapk3* and *Mapk2k1 (*genes encoding ERK1 and MEK1, respectively) remained unchanged (Figure 4(a)), suggesting that *de novo* transcription was not responsible for the elevated protein levels in activated cMVECs.

To address whether NOXs and transcription factors could be delivered to cMVECs with exosomes, we analyzed their presence in activated CD4^+^ T cells and CD4-exosomes. Although NOX2 and NOX4 were found in CD4^+^ T cells, they were not detected in the exosomes (Figure 4(c)). However, in contrast to NOXs, exosomes shed by CD4^+^ T cells were rich in MEK1/2 and ERK1/2 (Figure 4(c)). Furthermore, the uptake of PKH26-stained CD4-exosomes by cMVECs was confirmed by fluorescence imaging (Figure 4(d)). These data suggest that activated CD4^+^ T cells release MEK1/2 and ERK1/2 in exosomes, which can be taken up by cMVECs.

### 3.3. MEK/ERK Pathway Controls NOX4-Dependent Endothelial Dysfunction in Exosome-Activated cMVECs

**Figure 5:**
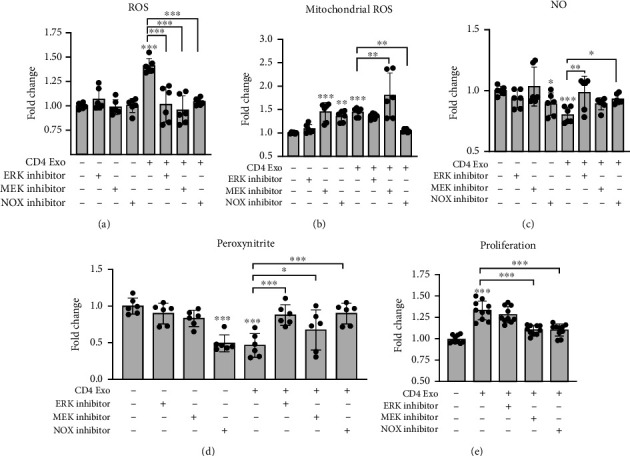
Oxidative stress induced in cMVECs by CD4-exosomes is regulated by ERK1/2 and MEK1/2 pathway. cMVECs were stimulated for 16 h with CD4-exosomes (CD4 Exo) in the presence or absence of ERK1/2 inhibitor (FR180204), MEK1/2 inhibitor (U0126), and NOX inhibitor (GKT136901). Panels (a)–(e) show effect of indicated treatment on total ROS levels (a), *n* = 6, mitochondrial ROS generation (b), *n* = 6, NO bioavailability (c), *n* = 6, peroxynitrite levels (d), *n* = 6, and cell proliferation (e), *n* = 10. ^∗^*p* < 0.05, ^∗∗^*p* < 0.01, and ^∗∗∗^*p* < 0.001 calculated by one-way ANOVA followed by Fisher's LSD post hoc test versus control group or for indicated groups.

In the next step, by using pharmacological inhibitors, we analyzed the contribution of NOXs and MEK-ERK pathways to regulate CD4-exosome-induced oxidative stress in cMVECs. We observed that NOX-1/4 inhibitor GKT136901 effectively blocked excessive ROS and superoxide production and reversed reduced NO levels triggered by CD4-exosomes (Figures 5(a)–5(c)). Inhibition of either MEK/ERK (with U0126) or ERK (with FR180204) also successfully protected cMVECs from increased ROS and reduced NO production and peroxynitrate levels but failed to suppress mitochondrial superoxide production in stimulated cells (Figures 5(a)–5(d)). Similarly, treatment with GKT136901 or U0126 reversed the enhanced proliferation of cMVECs triggered by CD4-exosomes (Figure 5(e)).

**Figure 6:**
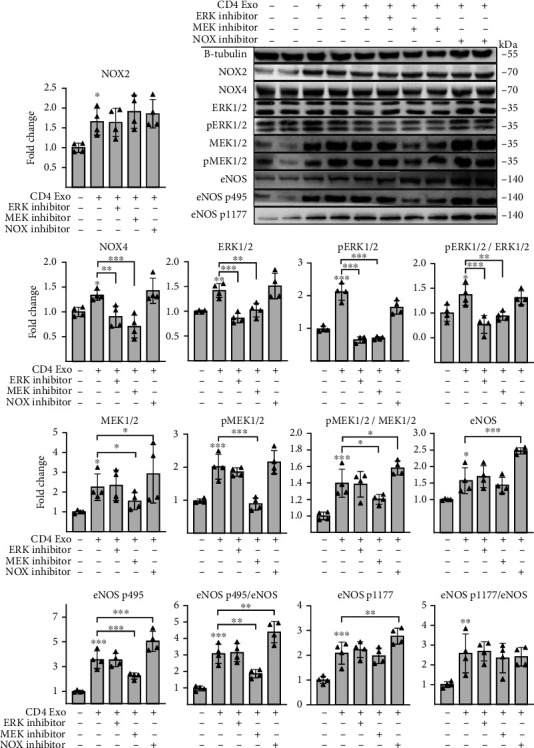
T cell-derived exosomes upregulate NOX4 in cMVECs through ERK1/2 and MEK1/2 pathway. This panel shows representative microphotographs of immunoblots and changes in levels of selected proteins in cMVECs in response to CD-4 exosomes in presence of ERK1/2 inhibitor (FR180204), MEK1/2 inhibitor (U0126), and NOX inhibitor (GKT136901). Cells were stimulated with exosomes for 16 h, *n* = 4. Protein levels were normalized to beta-tubulin, and phosphorylated proteins were additionally normalized to their respective nonphosphorylated forms. ^∗^*p* < 0.05, ^∗∗^*p* < 0.01, and ^∗∗∗^*p* < 0.001 calculated by one-way ANOVA followed by Fisher's LSD post hoc test versus control group or for indicated groups.

**Figure 7:**
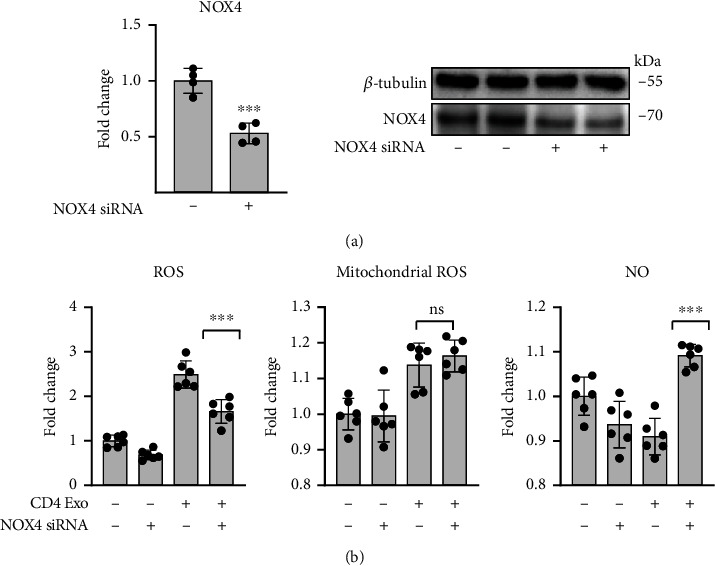
Oxidative stress caused by T cell-derived exosomes is mediated by NOX4. Panel (a) shows densitometry and representative immunoblots of cMVECs incubated with anti-*Nox4* siRNA for 36 h, *n* = 4. Panel (b) shows changes in total ROS, mitochondrial ROS, and NO levels in cMVECs transfected with anti-*Nox4* siRNA for 24 h and stimulated with CD4-exosomes for another 16 h, *n* = 6. ns-*p* > 0.05 and ^∗∗∗^*p* < 0.001 calculated by Student's *t*-test in (a) and one-way ANOVA followed by Fisher's LSD post hoc test versus indicated groups in (b).

As expected, ERK but also p-ERK/ERK levels were downregulated in cells treated with U0126 or FR180204 (Figure 6). As shown above, CD4-exosomes induced NOX4 production in cMVECs. We found that treatment with U0126 or with FR180204 effectively reduced NOX4 (but not NOX2) protein levels in cMVECs exposed to CD4-exosomes (Figure 6). Of note, protein levels of the MEK/ERK pathway as well as of NOX2 and NOX4 upregulated by CD4-exosomes were maintained in cMVECs treated with GKT136901 (Figure 6). To confirm the key role of NOX4 in oxidative stress in our model, we silenced *Nox4* in cMVECs prior treatments with CD4-exosomes. Silencing resulted in nearly 50% reduced NOX4 protein level (Figure 7(a)). Cells with the reduced NOX4 were protected from CD4-exosome-induced oxidative stress and showed elevated NO levels (Figure 7(b)). Collectively, these data point to NOX4 as a key enzyme in oxidative stress caused by CD4-exosomes and to MEK/ERK as a regulatory pathway.

### 3.4. Inflamed Hearts Contain Oxidative Stress-Inducing Exosomes

**Figure 8:**
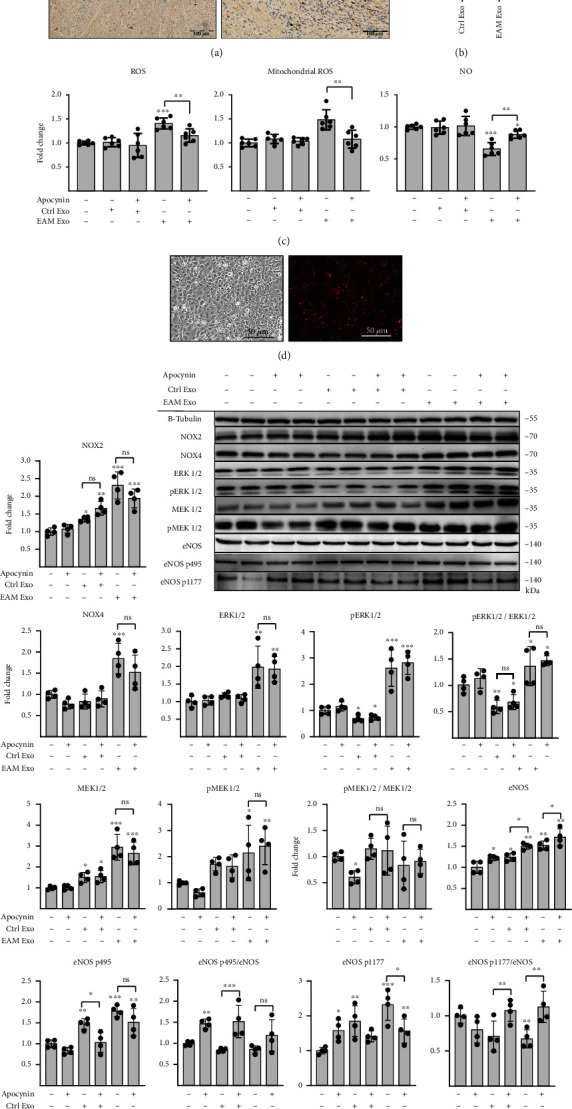
Exosomes isolated from inflamed hearts induce oxidative stress in cMVECs. Panel (a) shows representative microphotographs of CD3 staining in hearts of healthy controls and mice at day 19 of EAM. Scale bar = 100 *μ*m. Panel (b) represents quantification of exosomes isolated from hearts of healthy mice (Ctrl Exo) and hearts of mice at day 19 of EAM (EAM Exo), *n* = 6. Panel (c) shows relative changes in levels of total ROS, mitochondrial ROS, and NO in cMVECs in response to 16 h of incubation with Ctrl Exo or EAM Exo in presence or absence of apocynin (Apo), *n* = 6. Panel (d) shows representative microphotographs of PKH26-stained exosomes (red) bound to cMVECs after 16 h of incubation. Representative immunoblots and quantification of selected proteins in cMVECs stimulated for 16 hours with exosomes obtained from healthy or inflamed hearts are presented in panel (e). Protein levels were normalized to beta tubulin, and phosphorylated proteins were additionally normalized to their respective nonphosphorylated forms, *n* = 4. *p* > 0.05, ^∗^*p* < 0.05, ^∗∗^*p* < 0.01, and ^∗∗∗^*p* < 0.001 calculated by one-way ANOVA followed by Fisher's LSD post hoc test versus control group or for indicated groups.

In the next step, we analyzed whether cardiac inflammation is associated with generation of oxidative stress-inducing exosomes in vivo. Experimental autoimmune myocarditis (EAM) represents a model of CD4^+^ T cell-mediated cardiac inflammation, in which the heart is infiltrated with T lymphocytes during the acute inflammatory phase (d18-21, Figure 8(a)). We isolated exosomes from hearts of control and EAM groups at d19 (EAM-exosomes) and observed that inflammation was associated with an increased number of microvesicles in the cardiac tissue (Figure 8(b)). Treatment with exosomes (10^8^ particles/ml) obtained from healthy hearts showed no effect on ROS, NO levels, and mitochondrial superoxide production in cMVECs. In contrast, EAM-exosomes (10^8^ particles/ml) increased ROS and superoxide levels and reduced NO bioavailability in the treated cells, and these changes were reversed by apocynin (Figure 8(c)). Uptake of microvesicles isolated from EAM hearts by cMVECs was confirmed by fluorescence imaging (Figure 8(d)). In line with the CD4-exosome data, cMVECs treated with EAM-exosomes upregulated NOX2, NOX4, ERK1/2, MEK1/2, and eNOS protein levels (Figure 8(e)). Of note, this upregulation remained unaffected in the presence of apocynin, suggesting MEK/ERK activation, and the subsequent increase of NOXs was mediated by exosomes and independent of the induced oxidative stress in cMVECs.

## 4. Discussion

Secreted bioactive factors mediate mid- and long-distance cell-to-cell communication. Exosomes represent a specific class of signaling cues as they transport a mixture of proteins, nucleic acids, and other biomolecules [18]. Their cargo depends on the cell type they were shed off, and therefore, cell type-specific exosomes are expected to trigger unique responses. In this work, we specifically examined the role of exosomes shed by CD4^+^ T cells in the activation of cMVECs. Involvement of CD4^+^ T cells has been recognized in the pathogenesis of several cardiovascular diseases including atherosclerosis, myocardial infarction, and myocarditis [19–21]. It is well established that following activation of TCR, CD4^+^ T cells secrete a number of proinflammatory cytokines such as TNF-*α*, IL-6, IL-17, and others, which upregulate adhesive molecules in endothelial cells leading to tissue inflammation [22]. Unlike cytokines, CD4-exosomes do not modulate adhesive properties of endothelial cells suggesting that they are not transporting proinflammatory agents. This was a surprising finding, as oxidative stress has been often implicated in upregulation of adhesion molecules in endothelial cells. Thus, increased ROS was expected to induce adhesive properties of cMVECs. We can only speculate that either CD4-exosomes negatively regulate proadhesive mechanisms or ROS induces adhesion in preactivated endothelial cells only. In fact, prooxidative agents typically show pleiotropic effects and coactivate multiple molecular pathways; therefore, the exact mechanism of ROS-induced adhesion requires further investigation and may not be the same for all conditions. In line with our findings, infiltration of leukocytes into tissue induces oxidative stress [23]. Our *in vitro* results suggest that not only the secretome but specifically the exosomes shed by activated CD4^+^ T lymphocytes contribute to oxidative stress. Therefore, our results add exosomes to the list of bioactive factors secreted by CD4^+^ T cells and highlight them as potential important deregulators of ROS/NO balance in the inflammatory response in the heart. A growing body of evidence indicates that exosomes can modulate oxidative stress in recipient cells. For example, macrophage-derived exosomes can increase ROS production in neurons [24], microparticles derived from ischemic muscles, and blood platelets from septic patients induce oxidative stress in endothelial cells by increasing NOX levels and their activity [25] [26],^,^. Furthermore, exosomes have been associated with oxidative stress in human diseases. Exosomes containing miR-137 are elevated in Parkinson's disease and have been shown to induce oxidative stress in neurons [27]. Similarly, the serum of HIV infected patients is enriched in exosomes that carry proteins involved in oxidative stress and immune activation [28]. Importantly, not all types of exosomes induce oxidative stress. Published data demonstrate that exosomes derived from mesenchymal stem cells ameliorate ROS and inflammatory response [29–31]. Similarly, microvesicles derived of an activated T cell line were shown to protect human umbilical vein endothelial cells from actinomycin D-induced apoptosis by ameliorating ROS production [32].

**Figure 9:**
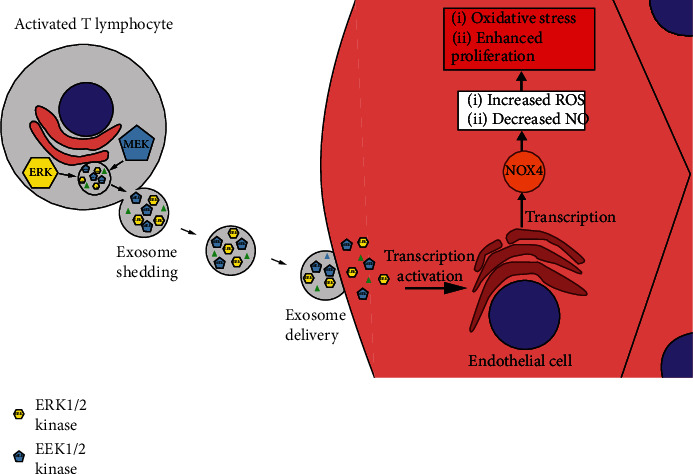
Schematic presentation of proposed mechanism. Activated T lymphocytes shed exosomes containing ERK1/2 and MEK1/2 kinases. These exosomes taken up by endothelial cells cause upregulation of NOX4 leading to oxidative stress and enhanced proliferation.

Exosomes can either up- or downregulate oxidative stress; therefore, it seems that their molecular cargo determines the ultimate effect in the recipient cell. In the case of CD4^+^ T cell-derived exosomes, we found no evidence for the presence of NOX2 and NOX4 in exosomes despite the presence of these enzymes in the activated CD4^+^ T cells. These data suggest a highly selective accumulation of cytoplasmic content into exosomes in CD4^+^ T cells. Unlike T lymphocytes, macrophage-derived exosomes were reported to contain functional NOX2 [24]. Instead, we detected ERK1/2 and MEK1/2 in exosomes, and these transcription factors are known to control NOXs levels in endothelial cells [33, 34]. As CD4^+^ T cell-derived exosomes transport ERK1/2 and MEK1/2 but not NOXs, we suggest that endothelial cells take up ERK1/2- and MEK1/2-rich exosomes and thereby increase their intracellular ERK1/2 and MEK1/2 levels. This leads to transcriptional upregulation of *Nox*s and eventually to oxidative stress (Figure 9). Consistent with this hypothesis, we observed a relatively quick (2 h after treatment with exosomes) increase in intracellular ERK1/2 and MEK1/2 protein levels, while the corresponding mRNAs remained unchanged. However, we cannot exclude an alternative mechanism that upregulates ERK1/2 and MEK1/2 in our experimental model.

Experiments with ERK1/2 and MEK1/2 inhibitors showed that the MEK-ERK pathway controlled ROS and mediated upregulation of specifically NOX4 and *Nox4* silencing confirmed the key role of this enzyme in exosome-induced oxidative stress. In the cardiovascular system, NOX4 generates superoxide constitutively, whereas NOX2 is an inducible enzyme [35]. It seems that NOX4 represents the most important NOX isoform in endothelial cells. In line with our findings, the expression of NOX4 in cardiomyocytes involved in mediating oxidative stress is responsible for the impairment of cardiac functions in a pressure overload model [35]. In endothelial cells, NOX4 appears to positively regulate functions related to angiogenesis. Increased levels of this enzyme promote proliferation, migration, and angiogenesis, which are connected to activation of ERK1/2 pathway [36].

Oxidative stress induced by exosomes enhanced mitochondrial ROS production, reduced NO bioavailability, and triggered proliferation of endothelial cells. It seems that elevated ROS levels due to upregulated NOXs affected normal mitochondrial metabolism and triggered mitochondrial ROS production. Such oxidative stress positive feedback mechanism is known as ROS-induced ROS release and has been described in endothelial cells [37]. Our data did not indicate the key involvement of NOX4 in mitochondrial ROS production; therefore, this process might be linked with NOX2 activity as described previously [38]. Elevated NOX2 levels were shown to be associated with increased antioxidant enzyme and eNOS protein levels. NOX2 was also shown to regulate the hemodynamic response to angiotensin II leading to endothelial dysfunction and increased mitochondrial ROS generation in aortic cells [39, 40]. ROS generation in mitochondria was originally thought to be a toxic by-product of ATP synthesis, whereas more recent views point to mitochondrial ROS as important signal transducers in regulating endothelial metabolism [41]. Nevertheless, a prolonged increment of ROS generation above basal levels can be detrimental and underlie the development of endothelial dysfunction, and atherosclerosis and cause hyperglycaemic damage [42, 43]. NO acts as a reversible inhibitor of the respiratory chain limiting mitochondrial activity and thus regulates respiration and ROS generation. In case of elevated systemic oxidative stress, NO reacts with excessive ROS forming peroxynitrite that irreversibly blocks multiple elements of the respiratory chain [44, 45]. Obtained data indicated that in our model, NO level was regulated by NOX4 and preserved by the addition of antioxidant apocynin. This suggests that CD4-exosomes trigger a reaction of NO with NOX4-derived superoxide radical and thereby reduce NO bioavailability in cMVECs. However, despite of increased ROS production, measured peroxynitrite levels in cMVECs were low. This unexpected result could be explained by enhanced processes utilizing peroxynitrite as a substrate, like SOD- or myeloperoxidase-dependent tyrosine nitration, or by enhanced peroxynitrite decomposition [46].

Cell damage is known to trigger apoptosis-induced compensatory proliferation, serving as a mechanism to maintain homeostasis during tissue regeneration [47]. Increased oxidative stress fuels this process through NOX-derived ROS, which activate pathways responsible for the proliferation [48]. In line with our data, in human dermal microvascular endothelial cells, NOX4- but not NOX2-derived ROS were shown to promote proliferation via ERK1/2 pathway [49].

Despite a straightforward effect in vitro, the actual contribution of CD4^+^ T cell-derived exosomes to endothelial dysfunction in vivo remains unclear. Currently, there are no available tools to specifically block exosome shedding or to modulate exosomal cargo in vivo to prove their impact on disease. Nevertheless, our findings could confirm the presence of pathogenic exosomes in inflamed hearts containing inflammatory CD4^+^ T lymphocytes; although, it should be acknowledged that these exosomes were not exclusively derived from CD4^+^ T cells. However, taking into account that myocarditis is associated with coronary microvascular dysfunction [50], exosomes produced by heart inflammatory cells should be considered as potential triggers of this pathogenic vascular condition. Furthermore, we believe that CD4^+^ T cell-derived exosomes could potentially contribute to the pathogenesis of other vascular diseases. For example, atherosclerosis represents a chronic inflammatory disease of arterial walls with the involvement of CD4^+^ T cells [19], and exosomes shed locally by these cells could exaggerate oxidative stress and promote endothelial dysfunction. However, more research is still needed to verify this hypothesis.

In conclusion, in this work, we identified exosomes as potentially important mediators in CD4^+^ T cell-dependent immune response, which can induce oxidative stress by delivering transcription factors. Specific neutralization of exosomal cargo might prevent from the induction of self-fuelling oxidative stress and endothelial dysfunction. Thus, the identification of a novel mechanism of intercellular communication opens up perspectives for the development of new therapeutic strategies for inflammatory cardiovascular diseases.

## Data Availability

The data used to support the findings of this study are available from the corresponding author upon reasonable request.

## Supplementary Materials

Supplementary Materials Supplementary figure 1: Effect of medium conditioned of activated CD4^+^ T lymphocytes and exosomes derived of resting CD4^+^ T lymphocytes on activation and oxidative stress in cMVECs. Panel (a) shows changes in membrane levels of adhesion molecules VCAM-1, ICAM-1, and p-selectin in cMVECs stimulated with medium conditioned of activated T lymphocytes (CM) or exosomes shed by resting CD4^+^ T lymphocytes (1 × 10^8^ particles/ml, rCD4 Exo) for 16 h, *n* = 6. In panel (b), influence of MVEC stimulation with CM or rCD4 Exo for 16 h on levels of total reactive oxygen species (total ROS), mitochondrial activity (mitochondrial ROS), and nitric oxide generation (NO) is shown, *n* = 6. ^∗∗∗^*p* < 0.001 calculated by one-way ANOVA followed by Fisher's LSD post hoc test versus control group.
