# Supplementary figures and images for: T Lymphocyte-Derived Exosomes Transport MEK1/2 and ERK1/2 and Induce NOX4-Dependent Oxidative Stress in Cardiac Microvascular Endothelial Cells
Source: Oxid Med Cell Longev. 2022 Sep 28;2022:2457687. doi: 10.1155/2022/2457687 (PMC9534701; doi:10.1155/2022/2457687)

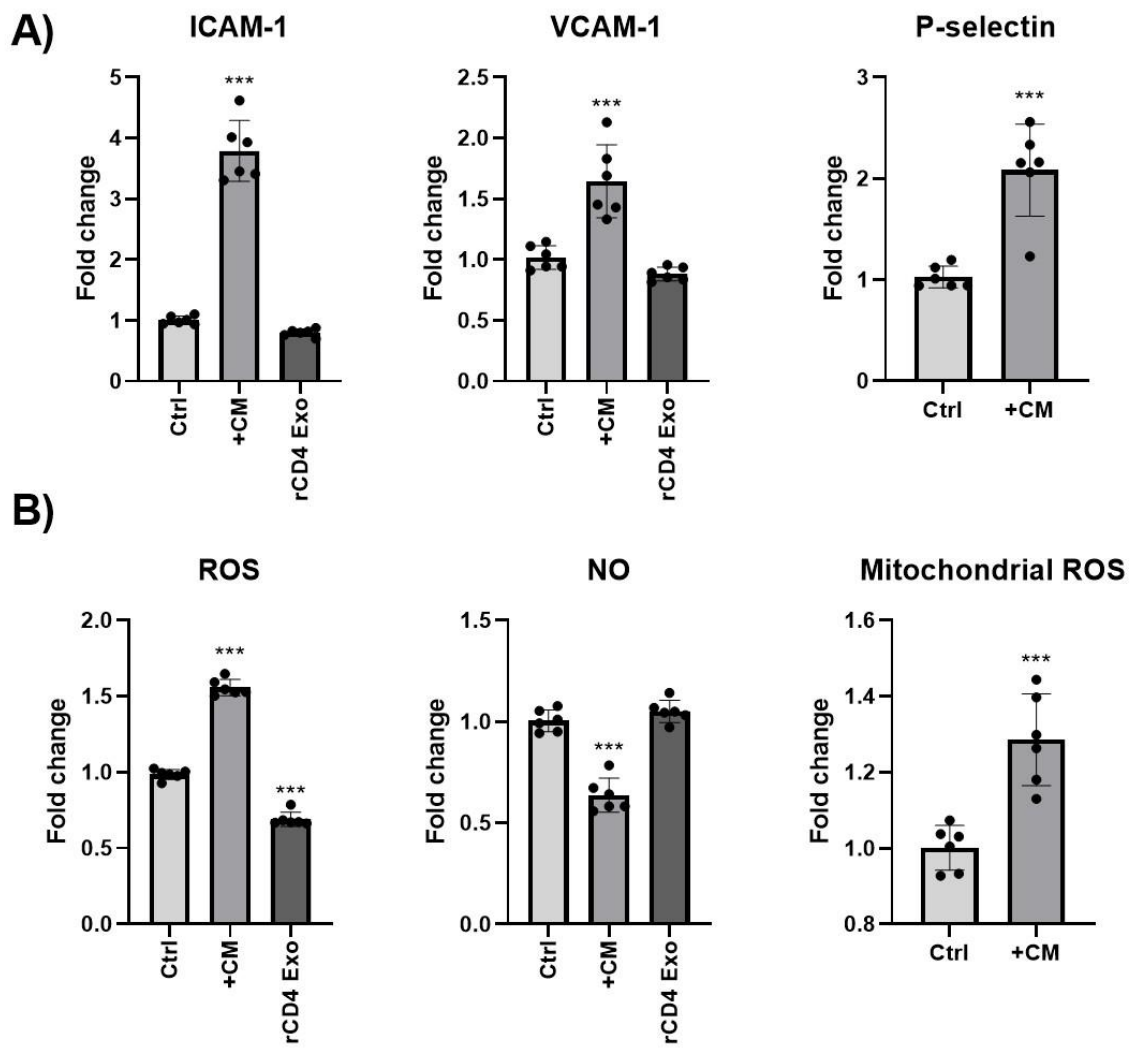

Supplement: Supplementary Materials — Supplementary figure 1: Effect of medium conditioned of activated CD4+ T lymphocytes and exosomes derived of resting CD4+ T lymphocytes on activation and oxidative stress in cMVECs. Panel (a) shows changes in membrane levels of adhesion molecules VCAM-1, ICAM-1, and p-selectin in cMVECs stimulated with medium conditioned of activated T lymphocytes (CM) or exosomes shed by resting CD4+ T lymphocytes (1 × 10^8^ particles/ml, rCD4 Exo) for 16 h, n = 6. In panel (b), influence of MVEC stimulation with CM or rCD4 Exo for 16 h on levels of total reactive oxygen species (total ROS), mitochondrial activity (mitochondrial ROS), and nitric oxide generation (NO) is shown, n = 6. ∗∗∗p < 0.001 calculated by one-way ANOVA followed by Fisher's LSD post hoc test versus control group. [file 2457687.f1.pdf]
